# Comparative Study of Carbon Nanosphere and Carbon Nanopowder on Viscosity and Thermal Conductivity of Nanofluids

**DOI:** 10.3390/nano11030608

**Published:** 2021-02-28

**Authors:** Thong Le Ba, Marcell Bohus, István Endre Lukács, Somchai Wongwises, Gyula Gróf, Klara Hernadi, Imre Miklós Szilágyi

**Affiliations:** 1 Department of Inorganic and Analytical Chemistry, Budapest University of Technology and Economics, Muegyetem Rakpart 3., 1111 Budapest, Hungary; bohus.mrc@gmail.com (M.B.); imre.szilagyi@mail.bme.hu (I.M.S.); 2 Centre for Energy Research, Institute for Technical Physics and Materials Science, Hungarian Academy of Sciences, Konkoly Thege M. út 29-33, 1121 Budapest, Hungary; lukacs.istvan@energia.mta.hu; 3 Department of Mechanical Engineering, Faculty of Engineering, King Mongkut’s University of Technology Thonburi, Bangmod, Bangkok 10140, Thailand; somchai.won@kmutt.ac.th; 4 National Science and Technology Development Agency (NSTDA), Pathum Thani 12120, Thailand; 5 Centre for Energy Research, Konkoly-Thege Miklós út 29-33, 1121 Budapest, Hungary; gyula.grof@gmail.com; 6 Department of Applied and Environmental Chemistry, University of Szeged, Rerrich Béla tér 1, 6720 Szeged, Hungary; hernadi@chem.u-szeged.hu; 7 Institute of Physical Metallurgy, Metal Forming and Nanotechnology, University of Miskolc, 3515 Miskolc-Egyetemváros, Hungary

**Keywords:** carbon nanosphere, carbon nanopowder, nanofluids, thermal conductivity, viscosity

## Abstract

A comparative research on stability, viscosity (µ), and thermal conductivity (k) of carbon nanosphere (CNS) and carbon nanopowder (CNP) nanofluids was performed. CNS was synthesized by the hydrothermal method, while CNP was provided by the manufacturer. Stable nanofluids at high concentrations 0.5, 1.0, and 1.5 vol% were prepared successfully. The properties of CNS and CNP nanoparticles were analyzed with Fourier-transform infrared spectroscopy (FT-IR), scanning electron microscope (SEM), X-ray photoelectron spectroscopy (XPS), specific surface area (S_BET_), X-ray powder diffraction (XRD), thermogravimetry/differential thermal analysis (TG/DTA), and energy dispersive X-ray analysis (EDX). The CNP nanofluids have the highest k enhancement of 10.61% for 1.5 vol% concentration compared to the base fluid, while the CNS does not make the thermal conductivity of nanofluids (k_nf_) significantly higher. The studied nanofluids were Newtonian. The relative µ of CNS and CNP nanofluids was 1.04 and 1.07 at 0.5 vol% concentration and 30 °C. These results can be explained by the different sizes and crystallinity of the used nanoparticles.

## 1. Introduction

Improvement of energy efficiency in many areas and applications is an indispensable part of the global sustainability plan [1]. For example, high-efficiency cooling, a technique that depends on heat exchangers, is an essential requirement in many applications such as air conditioning, automobile radiators, power plants, etc. [2]. Common fluids such as water, glycols, and oil are used as cooling liquids in the applications mentioned above [3]. However, they have some limited heat characteristics [4].

In 1993, Masuda et al. utilized the aluminum oxide nanoparticles (13 nm) in water base fluid. The obtained nanofluids had k enhancement of 30% [5]. In 1995, Choi et al. called the fluid containing nanoparticles as nanofluids first [6]. In 1999, a linear model of k and concentration was developed by Lee et al. with nanofluid containing copper oxide (CuO) and alumina (Al_2_O_3_) into a mixture of ethylene glycol and water [7]. Then, the experiments of Wang et al. showed k_nf_ was higher than the base fluids [8]. In 2001, multiwall carbon nanotubes (MWCNT) were added into the water for enhancing k up to 160% with 1% volume concentration [9]. The nanofluids have been potential materials with improved electrical, optical, and thermal characteristics [10]. During the last decades, there were numerous attempts to enhance k, convective thermal transfer coefficient, and thermal transfer rate by adding nanoparticles into the base fluid [11,12,13]. The property enhancement is dependent on the type, size, shape, and concentration of nanoparticles. Moreover, the nature and characteristic of base fluids such as pH, µ, and the presence of surfactants affect the increment of k [14,15]. However, the disadvantage of nanofluids is the stability issue because the nanoparticles tend to aggregate into larger particles and clusters. This limits the usability of nanofluids in heat transfer applications [16,17,18].

Several kinds of nanoparticles have been utilized in the preparation of nanofluids for enhancement of k, including metal (metal: Au, Ni, Cu, Ag; metal oxide: TiO_2_, ZnO, MgO, Fe_2_O_3_; metal carbides: TiC), carbon (diamond, carbon nanotube, graphene, graphene oxide, graphite, carbon dots [19]), and metalloid (SiO_2_, SiC) [20,21,22]. Some composite materials have been developed with enhanced k, such as Al/diamond, Al/SiC [23,24]. However, these materials have a high cost. Recently, liquids containing two or more nanoparticles, also called hybrid nanofluids, are under extensive research [25,26,27]. Due to the superior properties of the carbon-based nanoparticles, they are potential materials that can be utilized for many applications [3].

Generally, nanofluids can be produced with one of two methods: a single-step and two-step approach. For the first one, nanofluids are obtained by direct synthesis of the nanoparticles into the base fluid. In 2011, carbon/water nanofluid was prepared by Teng et al. by utilizing the plasma arc technique and showed that compared to water, k is enhanced by 25% at 50 °C [28]. Then, in 2013 a revised water-assisted synthesis system was utilized to produce carbon/water nanofluids [29]. In the two-step method, nanoparticles are produced first and then dispersed into the based fluid. The two-step method has been used mostly due to its simplicity and low cost [3].

The most important issue of nanofluids is stability, which affects their thermal properties and commercialization. While the thermal properties depend on a complex motion with a combination of agglomeration, Brownian movement, and thermophoresis effect, the stability is dependent on the interactions between the base fluid and the nanoparticles. These motions and interactions are affected by temperature [30], the properties of nanoparticles and base fluid [31], the pH of the medium [32], and the used surfactants [33]. In order to improve the stability of the nanofluids for the short term, the mechanical technique can be used, such as an ultrasound bath. In this method, the van der Waals attractive force is broken down between nanoparticles. This supports to disperse the nanoparticles better into the base fluids [34]. The surface modification and the particle size can enhance the repulsive force between the nanoparticles [35].

For the same materials, the values measured by different investigators were non-identical, mainly due to the preparation procedure and the morphology of agglomeration. One benchmark study was performed by Buongiorno et al. to compare k from different research groups. The samples were measured at various laboratories between 20 and 30 °C with some available methods, and the experimental error was obtained [36]. Moreover, the available theory does not explain the experimental results. The aggregation mechanism presents phonon transport from each massive particle to another one. This is affected by the size and shape of nanoparticles and the formed clusters [37,38].

The effect of concentration, temperature, and surfactants was studied on k increment by Estellé et al. [39]. Talaei et al. investigated the MWCNT and presented that the functional group concentration supports to increase the stability and k_nf_ [40]. The effects of the base fluid on k were reported by Nanda et al. [41] and Aravind et al. [42]. Chen et al. [43] and Nasiri et al. [44] studied k with the different structures of carbon nanotubes. Recently, Brzóska et al. [45] reported the thermal physical characteristics of long MWCNT.

A lot of attention has been paid to carbon spheres due to their utilizations, such as lubricants, catalyst supports, etc. [46]. Generally, carbon spheres can be prepared with three methods. First, carbon spheres may be produced directly as pyrolysis [47,48], chemical vapor deposition [49], and hydrothermal treatment [50]. The second method uses rigid templates as 3D macroporous silica [51], zeolite Beta beads [52]. Finally, carbon spheres can be obtained from the synthesized polymer spheres with thermal treatment [53,54]. Carbon nanospheres may be utilized in a lot of applications as photoluminescence [55], multiphoton bioimaging [56], anode materials in batteries [46], nanofluids [46,57], etc.

Among others, no study has been performed on the comparison of k_nf_ and µ_nf_ containing carbon nanomaterials with different sizes. In this paper, the carbon nanomaterial nanofluids based on water/ethanol are studied. The CNS is synthesized from cheap sugar, while CNP is supplied by the manufacturer. The particles were first analyzed with S_BET_, XPS, SEM-EDX, XRD, FTIR, and TGA. The nanofluids were prepared at three concentrations (0.5, 1.0, and 1.5 vol%), and their k_nf_ and µ_nf_ were measured at five temperatures (20, 30, 40, 50, and 60 °C).

## 2. Materials and Methods

### 2.1. Materials

Carbon nanospheres were prepared using the hydrothermal method [58]. Sucrose was put in an autoclave; then, the pH was set to 12 with NaOH solution. Under autogenous pressure at 180 °C, the mixture was treated for 12 h. The reaction’s product was washed three times with distilled water then suspended in a 45% ethanol-water solution. The suspension was centrifuged at 4000 rpm for 20 min. The settled material was filtered and dried at 70 °C after washing with warm distilled water overnight. The product was a brown powder [59].

Carbon nanopowders, base fluids (ethylene glycol and ethyl alcohol), surfactants (Gum Arabic (GA), Triton X-100, and Tween 80 (T80) were purchased from Sigma Aldrich (Saint Louis, MO, USA).

### 2.2. Preparation of Nanofluid

The stability of nanofluids was investigated by using different kinds of solvents and surfactants. Various ratios of the solvent mixture were considered as well, and in the following, we report about only those configurations that were observed as stable nanofluids.

The nanofluids were obtained by adding CNS and CNP into the base fluid of deionized water (DI)/ethanol and DI/ethylene glycol, respectively. The ratio of DI and ethanol was 5:1. In the case of DI and ethylene glycol, the ratio was 1:1, and T80 was used as a surfactant with a concentration of 3.3 vol%. For CNP, the different base fluids were investigated; however, the nanofluids without surfactant were not stable. The volume fraction of nanoparticle content was 0.5, 1.0, and 1.5%. The CNS and CNP nanofluids were sonicated using an ultrasonication instrument for 1 h at 130 W and 45 kHz. The prepared nanofluids were stable for several days.

### 2.3. Characterization Techniques

The morphology analysis of nanoparticles was completed by LEO 1440 XB SEM (LEOGmbH, Oberkochen, Germany) at 5 kV with a secondary electron detector in a high vacuum mode.

The chemical components of CNS and CNP were examined by utilizing EDX analysis with a JEOL JSM-5500LV electron microscope (Tokyo, Japan) and XPS with X-ray photoelectron spectrometer (Berlin, Germany) having 150 W (14 kV) X-ray source. The investigation on the crystallinity of the CNS and CNP was performed by using Panalytical X’PERT PRO MPD XRD with Cu K_α_ irradiation, resolution of 3 degrees/min, and the 2*θ* range of 5° to 65°. The FTIR spectroscopy of CNS and CNP was performed by Excalibur FTS 3000 BioRad FTIR (Bio-Rad, Digilab, UK) in the wavenumber range of 400–4000 cm^−1^ and transmittance mode. The sensitivity was 4 cm^−1^, and the number of scans was 64.

According to the multipoint Brunauer–Emmett–Teller technique, the S_BET_ of CNS and CNP was determined by utilizing nitrogen adsorption isotherms at −196 °C.

The effect of temperature on the nanoparticles was studied by utilizing an STD 2960 TG/DTA (TA Instruments Inc., New Castle, DE, USA) instrument with a heating rate of 10 °C/min. The temperature range was from room temperature to 800 °C. The experiments were performed in air.

The stability of CNS and CNP nanofluids was tested by using an Avantes AvaSpec-2048 Fiber Optic spectrometer (Avantes BV, Apeldoorn, Netherlands). After a period, 20 µL of each sample was diluted with 2 mL of DI, and their maximum absorbance was recorded.

The rheology of CNS and CNP nanofluids was investigated with three replicas using an Anton Paar Physica MCR 301 (Anton Paar, Ashland, VA, USA) rotation viscometer at 15 different shear rates and five temperatures. The amplitude was 5%. The range of angular frequency was 100 to 2000 s^−1^.

Based on the modified transient plane source approach, an SKZ1061C TPS Thermal Conductivity Tester (SKZ Industrial, Shandong, China) was used for measuring k of CNS and CNP nanofluids. All nanofluids were measured three times at five temperatures (20, 30, 40, 50, and 60 °C). The mean value and standard error were calculated for use in the figures. In order to increase the temperature of the nanofluids at the defined setpoint, a temperature-controlled oven was used.

## 3. Results and Discussion

### 3.1. Structure of CNS and CNP

**Figure 1:**
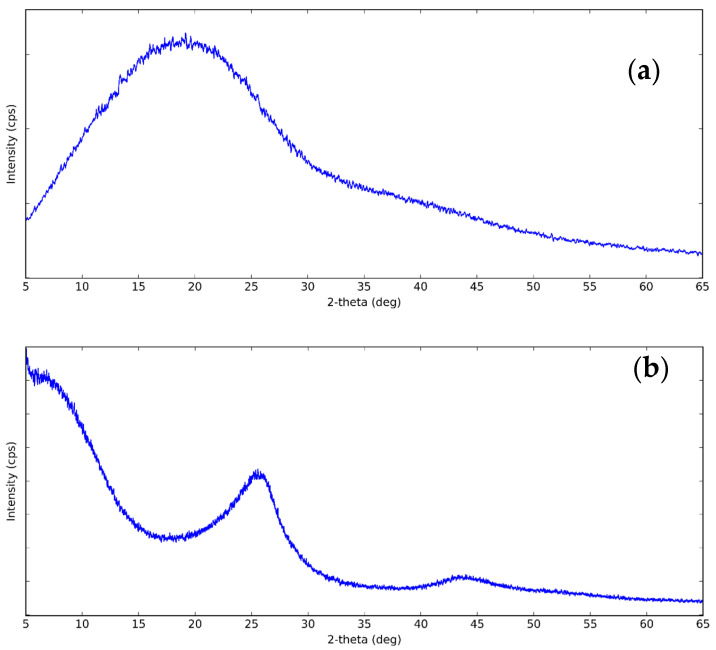
XRD pattern of (**a**) carbon nanosphere (CNS) and (**b**) carbon nanopowder (CNP) at the following XRD conditions: X-Ray: 40 kV, 30 mA. Scan speed: 3.0 degree/min.

The XRD pattern of CNS and CNP is shown in Figure 1. The figure showed that the structure of CNS was amorphous due to the single broad diffraction peak centered at 20°. For CNP, it can be seen that there are two broad peaks at 2θ = 25° and 2θ = 43.8°. The diffraction peaks correspond to the (002) and (101) planes of graphite. The crystallinity in CNP is higher than in CNS because of the higher degree of graphitization [60]. This can increase the thermal conductivity of CNP nanofluids due to the amorphous particles scatter phonon [61].

**Figure 2:**
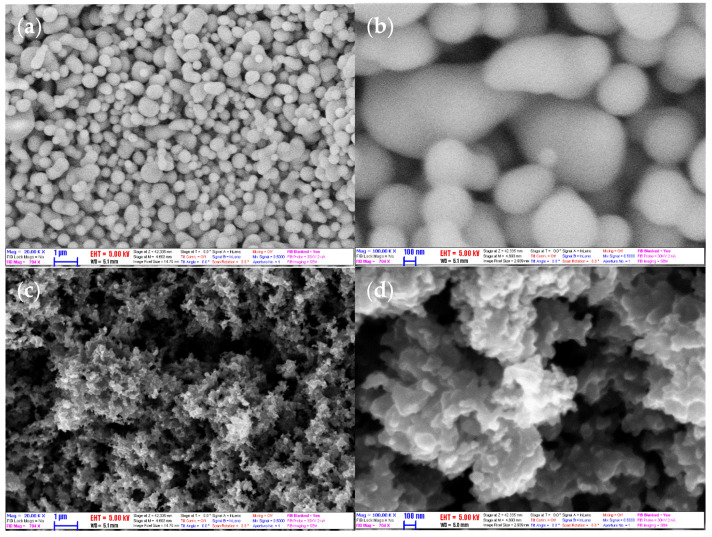
SEM images of (**a**,**b**) CNS and (**c**,**d**) CNP with magnification ×20,000 and ×100,000.

Figure 2 shows the SEM photographs of the morphology of CNS (a, b) and CNP (c, d). From the figure, it can be seen that the CNS was uniformed in a well-shaped sphere with a smooth surface, while the CNP was irregular; and the particles of both types tend to aggregate. By treatment of the SEM images, the average diameter of the particles was obtained. The diameter of CNS and CNP was 198 and 60 nm, respectively. The size of the CNP is in agreement with the manufacturer (<100 nm). The size of the CNP is smaller than the CNS’s. This supports CNP nanofluids with higher k. However, the attractive force between CNP particles is larger, due to that most of the particles bond together to form a cluster; it makes the CNP nanofluid challenging to stabilize.

The nanoparticle shape strongly affects the transport processes in nanofluids [62], resulting in that the thermal conductivity and viscosity do not vary only on the volume fraction of nanoparticle, but in a different extent rely on the particle shape. As the molecular level interactions take place at the particle surface, the particle shapes impact the thermal and momentum transfer. The particle shape impacts on the particle–particle interaction (e.g., collision) and in the particle–fluid interaction (e.g., liquid layering) as well. These complex static and dynamic processes results in differences in augmentation, in the boundary (hydraulic and temperature) layer formation, in the role of radiation of the heat transfer, and in the liquid layering.

**Figure 3:**
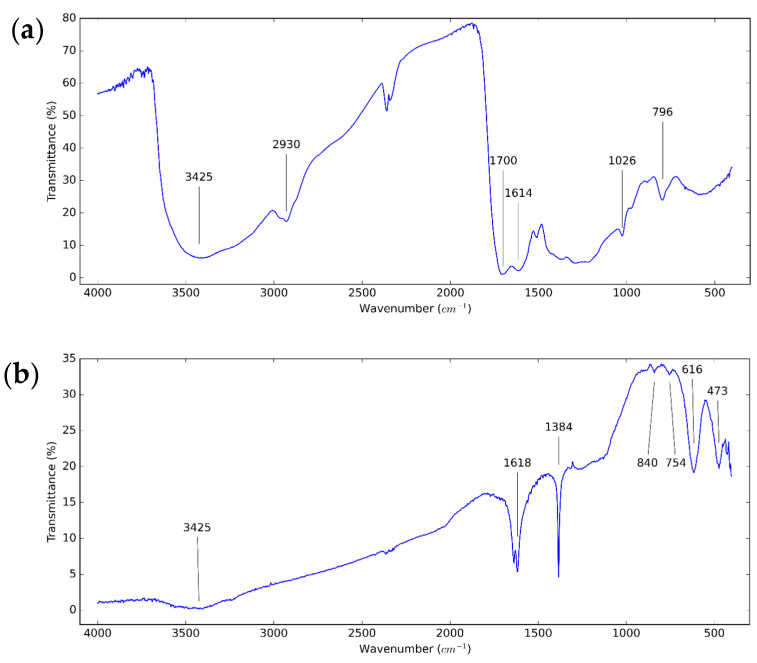
FT-IR spectrum of (**a**) CNS and (**b**) CNP dry particles.

In order to investigate the functional groups of the used CNS and CNP to select the type of solvents and surfactants, FT-IR analysis was performed. The FT-IR spectrum of the used nanoparticles is shown in Figure 3.. For both types of nanoparticles, the property of vibration of –OH groups is at 3425 cm^−1^. At 1614 cm^−1^ and 1618 cm^−1^, the characteristic peak of the C=C double bonds is observed. The peaks at around 754, 796, and 840 cm^−1^ are caused by the hydrogen wagging absorption of aromatic rings [63]. For the CNS, the active mode at around 2930 cm^−1^ refers to the vibration of C–H bonds [60]. The peak at 1700 cm^−1^ and 1026 cm^−1^ is assigned to the –OH and C–O bonds, respectively [64]. For the CNP, the fingerprint bands at 616 and 473 cm^−1^ present the aromatic structure with monosubstitution [65].

The chemical composition of the CNS and CNP was obtained by EDX. The main elements, including C and O (H cannot be observed) from EDX and XPS measurements and S_BET_ are presented in Table 1. The table presents the average of the atomic percentage at various measurement points. The atomic percent of the oxygen content in the CNP was lower than in the CNS. The CNP has 91.6 atomic percent of carbon from EDX analysis. The S_BET_ of CNP is 10 times higher than that of CNS.

**Figure 4:**
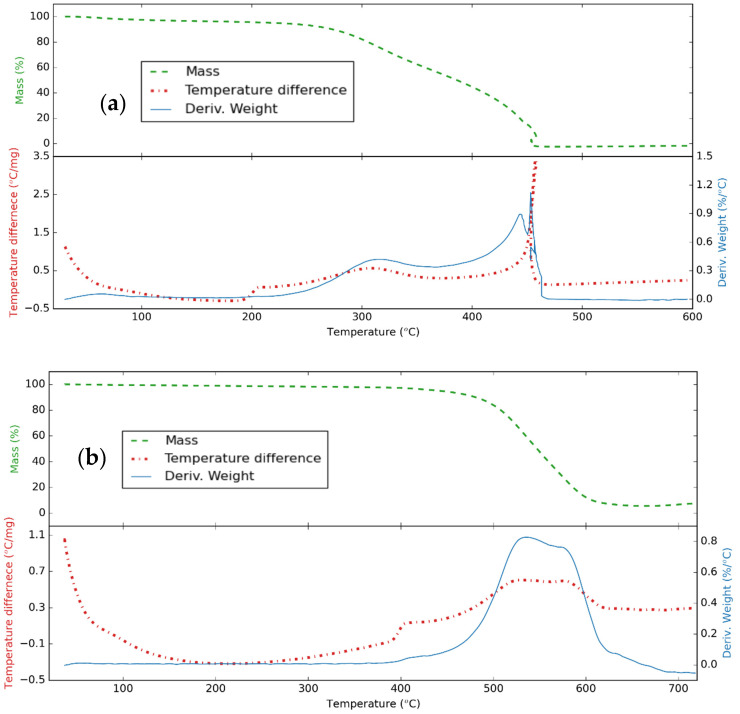
Thermal analysis curve for (**a**) CNS and (**b**) CNP with a heating rate of 10 °C/min in airflow.

Figure 4 presents the thermal analysis of the CNS and CNP samples in air. For the CNS, there are two stages of weight loss observed. The first one refers to the removal of the absorbed water, the dehydration of the functional groups, and the densification of the surface layer of the CNS to 245 °C, and the weight loss is around 6.3%. The next one can be attributed to the oxidation of the carbonaceous nanomaterials to 450 °C [66]. For the CNP, the first stage lasts to 425 °C, and the weight loss is 3.9%; then, the oxidizing process happened to 650 °C [67]. The remaining ash from the oxidization was 0.5% and 5.5% for the CNS and CNP, respectively. This indicates that the structure of the CNP is more durable than that of CNS. This happened because the carbon atoms were oxidized more in the CNS nanoparticles. Similarly, reduced graphene oxide is highly stable compared to pure graphene oxide [68].

**Figure 5:**
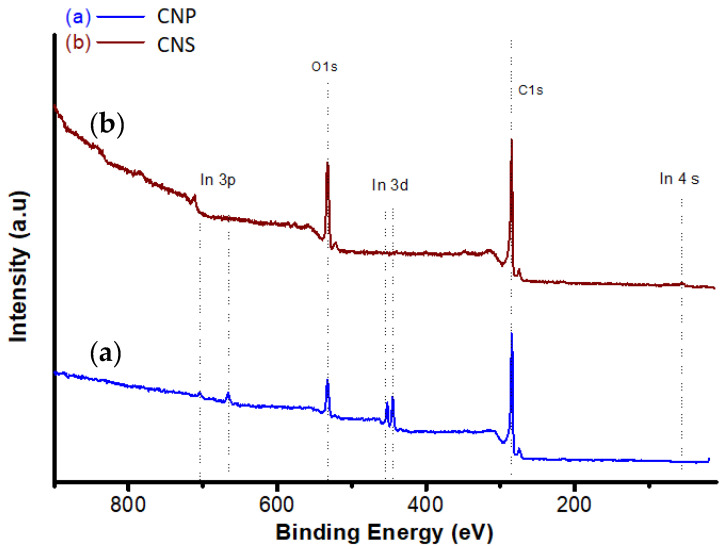
XPS analysis for (**a**) CNP and (**b**) CNS with 40 eV pass energy and 0.3 s dwell time.

**Figure 6:**
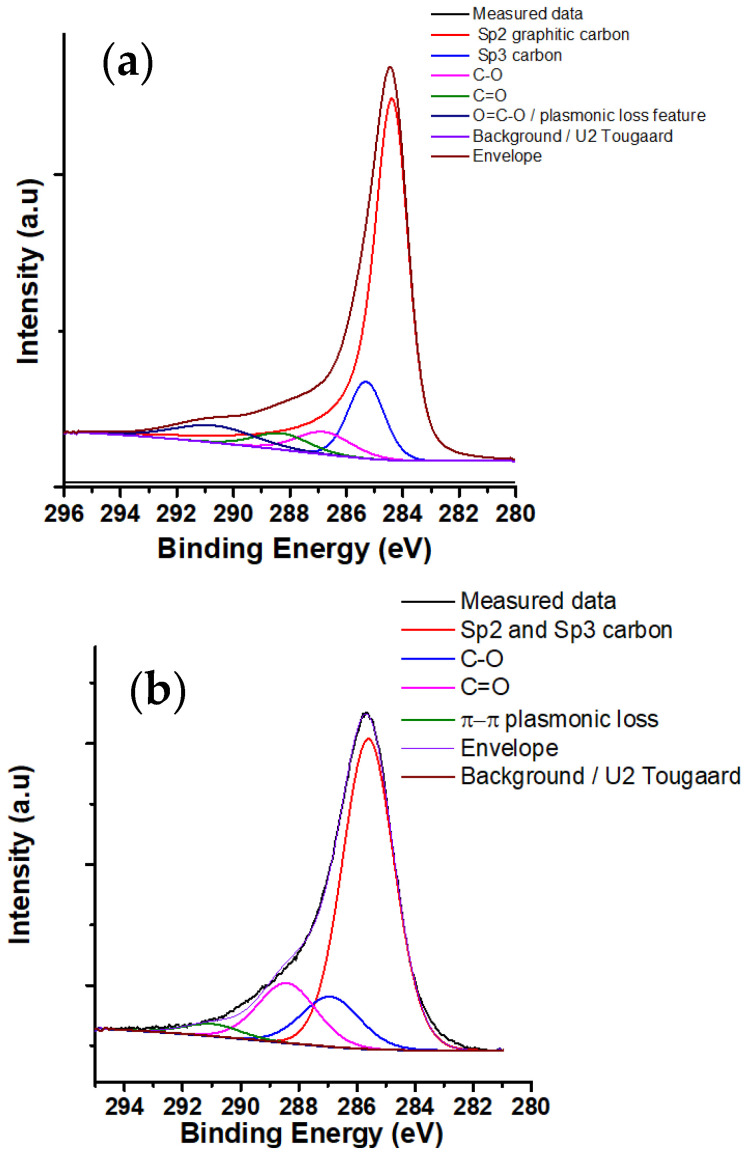
C 1s XPS spectrum of (**a**) CNP and (**b**) CNS.

XPS spectrum and the deconvolution of the carbon 1s of CNS and CNP are shown in Figure 5 and Figure 6. From the figures, the carbon 1s contains five components: sp^2^, sp^3^, C–O, C=O, and O–C=O/loss feature observed at 284.3, 285.3, 286.8, 288.3, and 290.7 eV, respectively [69]. At 290.7 eV, the peak can be the mixture of plasmonic loss feature and carbon peak. Table 2 shows the concentration of chemical bonds from XPS measurements. The percentage of O–H in CNS is greater in CNS than in CNP. Also, the percentage of C–O and C=O of CNS is higher than that of CNP. This is consistent with the results of S_BET_. With greater carbon/oxygen ratio, the S_BET_ values of particles are higher [70].

**Figure 7:**
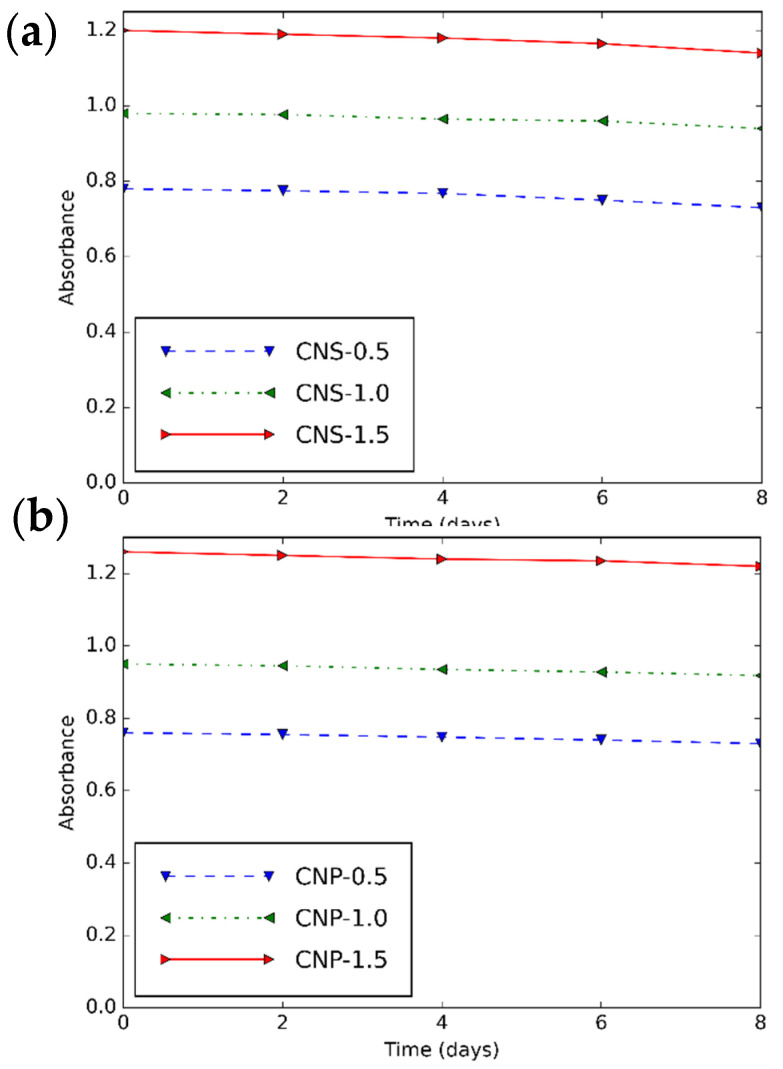
Maximum absorbance—time diagram of (**a**) CNS and (**b**) CNP nanofluids for different concentrations.

The maximum absorbance of CNS and CNP nanofluids over days is shown in Figure 7. It is obvious that the prepared nanofluids are stable for 4 days.

### 3.2. Rheological Characteristics of CNS and CNP Nanofluids

**Figure 8:**
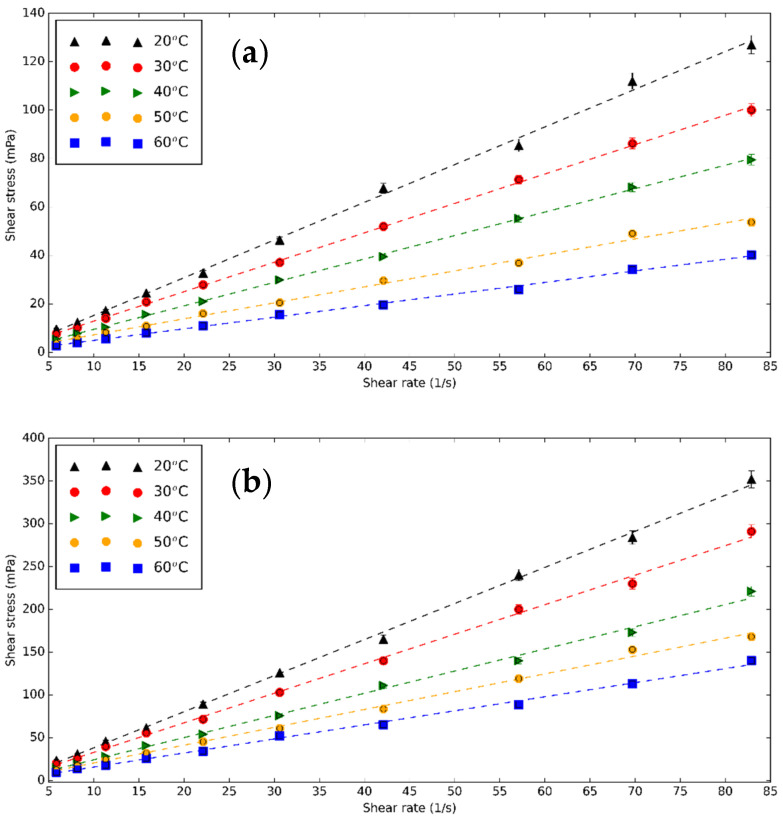
Shear stress—shear rates diagram of (**a**) CNS and (**b**) CNP nanofluids for 0.5 vol% at different temperatures.

One of the most important factors determining the quality of the heat transfer fluid is µ because the increase of µ causes higher pumping energy. Similar to other fluids, temperature affects µ_nf_. The viscometer is calibrated with DI and the measured value matches closely the theoretical result. The rheological measurements are performed on base fluid, CNS, and CNP nanofluids for three concentrations of 0.5, 1.0, and 1.5 vol% at different temperatures. Figure 8 presents the shear stress of 0.5 vol% CNS and CNP nanofluids as a function of shear rate at different temperatures. Increasing temperature makes the shear stress of the nanofluids lower due to the Brownian motion, while the concentration of CNS and CNP increases the shear stress [71,72]. The nanofluids are Newtonian because the chart line can be considered as linear.

The surface to volume ratio is significantly higher (see Figure 2) for CNP compared to the CNS. Forming nanofluid with the same concentration results more particle–fluid interaction resulting in higher shear stresses and higher viscosity.

**Figure 9:**
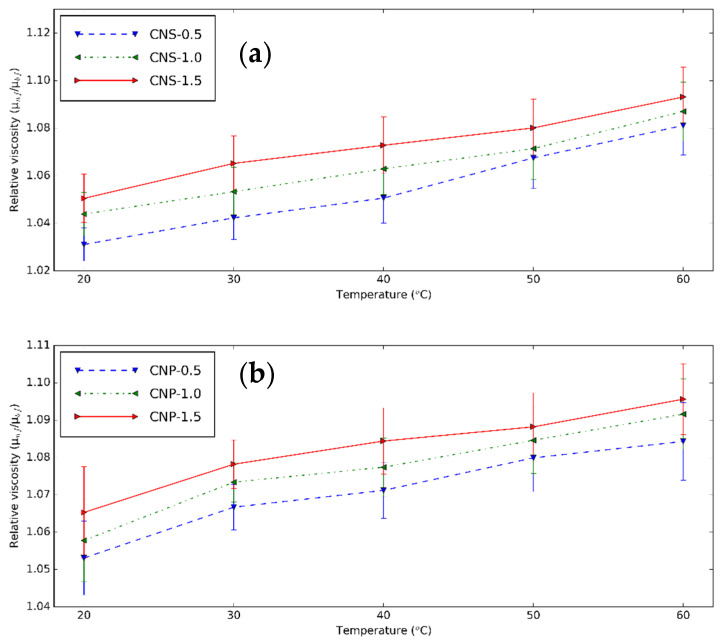
Relative viscosity–temperature diagram of (**a**) CNS and (**b**) CNP nanofluids for different concentration at different temperatures.

The relative µ of CNS and CNP nanofluids, which is the ratio of the µ of nanofluids and base fluid, is shown in Figure 9 from 20 to 60 °C. The nanoparticle content increases µ. This is probably due to the formed clusters [3]. With temperature, µ_nf_ increases faster than that of the base fluid. The similar relative µ_nf_ are obtained for the two particles. However, the µ of CNP nanofluid (at 0.5 vol% at 20 °C, 3.911 mPas) is much higher than that of the CNS nanofluids (1.545 mPas) due to the usage of surfactant [73]. The µ increment of CNS nanofluid is between 3.11% and 9.31%, while this range of the CNP nanofluids is 5.31% and 9.56%.

**Figure 10:**
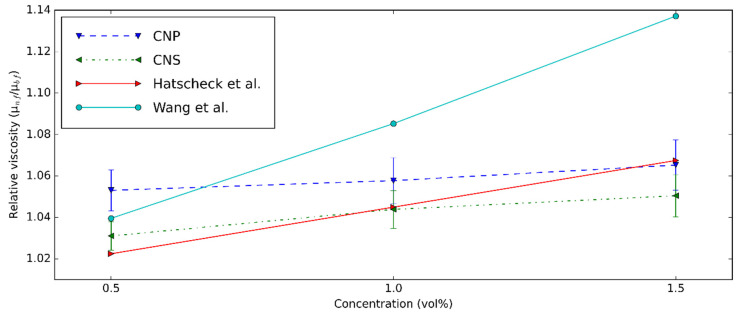
Relative viscosity comparison between the present study and the result of Hatscheck et al. [74] and Wang et al. [8] at 20 °C and different concentrations.

The relative µ obtained in the current study was collated with the values published in the previous investigations to confirm the results [8,74]. The collation was performed with different concentrations and at 20 °C. Figure 10 shows the comparison between the present study and the previous results. This study has a result similar to the predicted values from Hatscheck et al. [74] and Wang et al. [8] at 0.5 vol%, while at higher concentrations, the Wang formula [8] overestimated the prepared nanofluid viscosities.

### 3.3. Thermal Conductivity of CNS and CNP Nanofluids

**Figure 11:**
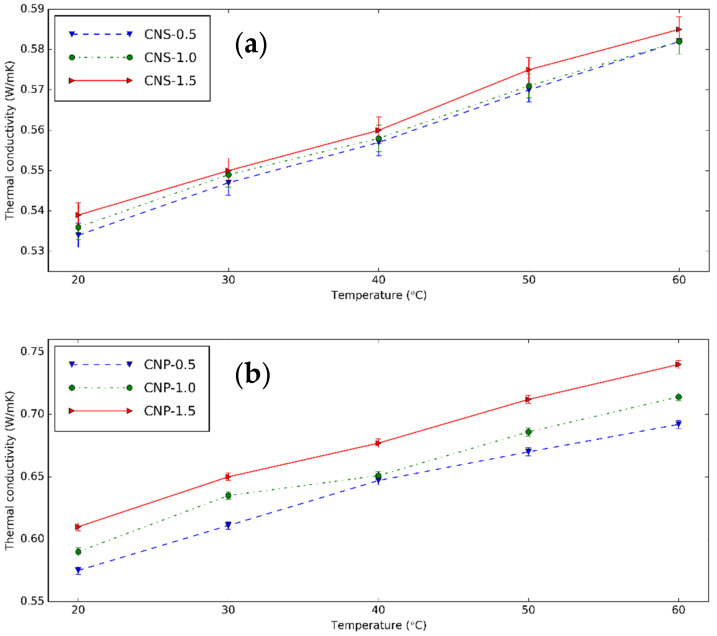
k of (**a**) CNS and (**b**) CNP nanofluids with different concentrations at different temperatures.

**Figure 12:**
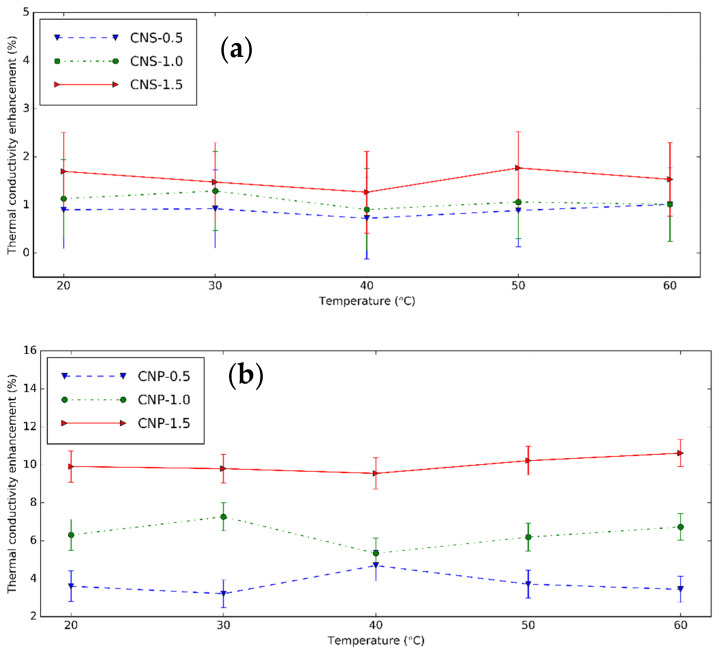
k enhancement of (**a**) CNS and (**b**) CNP nanofluids with different concentrations at different temperatures.

The k and its increment of CNS and CNP nanofluids is shown in Figure 11 and Figure 12 at different temperatures. The used device is trustable with a low error when k of distilled water was measured. The nanofluids have a higher k than these fluids without nanoparticles at experimental temperatures. Temperature increases k_nf_ because of the Brownian movement of nanoparticles. At 0.5 vol%, from 20 to 60 °C, k increases from 0.534 to 0.582 W/mK or 8.99% increment for CNS, and from 0.575 to 0.692 W/mK or 20.35% for CNP. The higher number of nanoparticles dispersed in the nanofluids or concentration of the nanofluids makes k greater. At 30 °C, from 0.5 vol% to 1.5 vol%, k increases by 0.55% for CNS and 6.38% for CNP nanofluids. In literature, from the results of Mirsaeidi et al. [19], the carbon dots nanofluids have k of 0.261 and 0.27 W/mK, the k enhancement of 3.6% and 7.1% for 0.4 and 1.0 vol%. Brzóska et al. [45] showed the k increments of MWCNT nanofluids were 15.4% and 29.3% for 0.5 and 1.0 vol%. Compared to the carbon dot nanofluids, the CNP nanofluids have a similar result, while the CNS nanofluids have lower k enhancement.

The k enhancement of CNP nanofluids is greater than that of the CNS nanofluids. This occurs due to the smaller sizes and higher crystallinity of the CNP nanoparticle. Moreover, based on the XPS analysis, CNP has higher carbon/oxygen ratio, this improves better the k of the CNP nanofluids [70]. It is concluded that although the CNP nanofluids are difficult for stabilization, these nanofluids have better k. The carbon dots with smaller size improve more k for the nanofluids based on them [19]. These results are lower than the k enhancement of MWCNT nanofluids [45]. This can be explained by the long shape of MWCNT.

**Figure 13:**
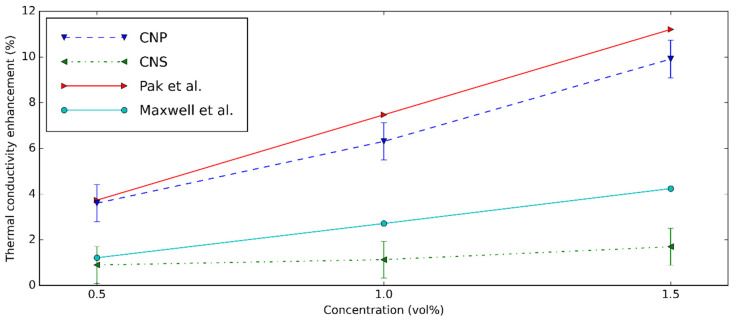
k enhancement comparison between the present study and the result of Maxwell et al. [76] and Pak et al. [75].

The comparison of k enhancement between this study and the previous research is shown in Figure 13. Clearly, the results estimated from Pak’s model [75] match with CNP nanofluids, while the Maxwell model [76] is suitable for the CNS nanofluids.

### 3.4. Regression Correlations

According to the research of Azmi et al. [77] and based on the mean values of the measured data, the following correlations are developed:

(1) $$\mathrm{Relative}\;\mathrm{viscosity}=\frac{\mu_{\mathrm{nf}}}{\mu_{\mathrm{bf}}}=1.029{\left(1+\frac{T}{60}\right)}^{0.08543}{\left(1+\frac{\phi }{100}\right)}^{1.379}{\left(1+\frac{d}{60}\right)}^{-0.01628}$$

(2) $$k_{\mathrm{nf}}=k_{\mathrm{bf}}\times \;1.102{\left(1+\frac{T}{60}\right)}^{0.00192}{\left(1+\frac{\phi }{100}\right)}^{1.142}{\left(1+\frac{d}{60}\right)}^{-0.06916},$$

where φ, d, and T are volume concentration, particle size, and temperature.

**Figure 14:**
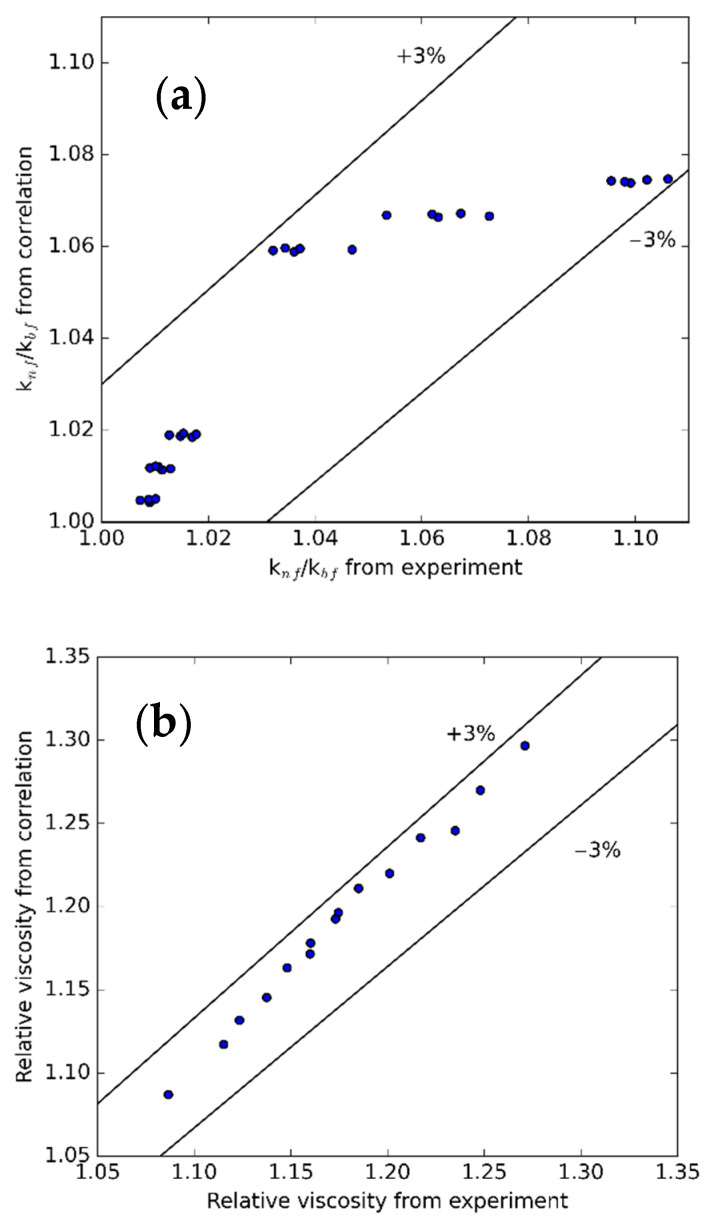
Comparison between (**a**) k and (**b**) µ obtained from experiment and proposed correlation.

Figure 14 presents the comparison of the k and µ from the measurement and regression equations. The average and standard deviations are 0.30% and 0.37% for µ; 1.04% and 0.74% for k. With these results, it can be proved that these correlations are applicable for future applications.

## 4. Conclusions

The CNS and CNP nanofluids were successfully prepared by using ultrasound and surfactants. The comparative research on stability, µ, and k was performed for the first time. The CNS and CNP nanoparticles were studied with XPS, S_BET_, FTIR, XRD, SEM-EDX, TG/DTA. The results present that the structure of CNP is more durable than that of CNS. While the uniformity of CNS is higher, the size of CNS is larger. The nanofluids with high concentrations can be considered as stable for at least 4 days.

The CNP nanofluids have the highest k enhancement of 10.61% for 1.5 vol% concentration compared to the base fluid, while the CNS does not make k_nf_ significantly higher. These nanofluids were Newtonian. The relative µ of CNS and CNP nanofluids was 1.04 and 1.07 at 0.5 vol% concentration and 30 °C. With CNP nanoparticles, the T80 surfactant was used for stabilizing the nanofluids. It makes the µ of CNP nanoparticles much higher. Based on the measured data, the regression correlations were proposed for future usage.

## Figures and Tables

**Table 1:** S_BET_, XPS and EDX results of CNS and CNP dry particles.

Nanoparticles	S_BET_	Atomic Percent (XPS)		Atomic Percent (EDX)	
	(m^2^/g)	C	O	C	O
CNS	9	76.9	23.1	81.4	18.6
CNP	106	90.7	9.3	91.6	8.4

**Table 2:** Concentration of chemical bonds on the surface of CNS and CNP from XPS.

Nanoparticles	C 1s (%)					O 1s (%)	
	sp^3^	sp^2^	C–O	C=O	O–C=O	C=O	O–H
CNP	72.1	12.3	5.5	4.0	6.1	35.4	64.6
CNS	69.3		12.6	15.0	3.1	8.2	91.8

## Data Availability

The data presented in this study are available on request from the corresponding author.
